# Criteria-based audit on management of eclampsia patients at a tertiary hospital in Dar es Salaam, Tanzania

**DOI:** 10.1186/1471-2393-9-13

**Published:** 2009-03-27

**Authors:** Hussein Lesio Kidanto, Ingrid Mogren, Siriel N Massawe, Gunilla Lindmark, Lennarth Nystrom

**Affiliations:** 1 Department of Obstetrics and Gynaecology, Muhimbili National Hospital, Dar es Salaam, Tanzania; 2 Department of Clinical Science, Obstetrics and Gynaecology, Umeå University, Umeå, Sweden; 3 International Maternal and Child Health, Department of Women's and Children's Health, University of Uppsala, Sweden; 4 Department of Public Health and Clinical Medicine, Epidemiology and Public Health Sciences, Umeå University, Umeå, Sweden

## Abstract

**Background:**

Criteria-based audits have been used to improve clinical management in developed countries, but have only recently been introduced in the developing world. This study discusses the introduction of a criteria-based audit in a tertiary hospital in an African setting, assesses the quality of care among eclampsia patients and discusses possible interventions in order to improve the quality of care.

**Methods:**

We conducted a criteria based audit of 389 eclampsia patients admitted to Muhimbili National Hospital (MNH), Dar es Salaam Tanzania between April 14, 2006 and December 31, 2006. Cases were assessed using evidence-based criteria for appropriate care.

**Results:**

Antepartum, intrapartum and postpartum eclampsia constituted 47%, 41% and 12% of the eclampsia cases respectively. Antepartum eclampsia was mostly (73%) preterm whereas the majority (71%) of postpartum eclampsia cases ware at term. The case fatality rate for eclampsia was 7.7%. Medical histories were incomplete, the majority (75%) of management plans were not reviewed by specialists in obstetrics, specialist doctors live far from the hospital and do not spend nights in hospital even when they are on duty, monitoring of patients on magnesium sulphate was inadequate, and important biochemical tests were not routinely done. Two thirds of the patient scheduled for caesarean section did not undergo surgery within agreed time.

**Conclusion:**

Potential areas for further improvement in quality of emergency care for eclampsia relate to standardizing management guidelines, greater involvement of specialists in the management of eclampsia and continued medical education on current management of eclampsia for junior staff.

## Background

It is estimated that every year eclampsia is associated with about 50 000 maternal deaths worldwide, most of which occur in developing countries[1,2]. The incidence of eclampsia is higher in developing countries (1 in 100–1700 deliveries) than in developed countries (1 in 2000 deliveries). This is probably due in particular to pregnant women's lack of easy access to appropriate antenatal care in those settings. Even in countries with low maternal mortality, a substantial proportion of the maternal deaths will be attributed to pre-eclampsia/eclampsia. In the United Kingdom, pre-eclampsia and eclampsia account for 15% of the direct maternal deaths and two-thirds are related to pre-eclampsia [3].

Although eclampsia remains a cause of maternal and foetal morbidity and mortality in developed countries, the incidence has fallen considerably in developed countries due to high quality antenatal care [4,5]. However, this decline is not yet observed in developing countries [6,7].

Criteria-based audits (CBA) have been used to improve clinical management in developed countries, but have only recently been introduced in developing countries. CBA compares current practices against written and agreed upon criteria. The selected criteria are based on evidence-based care and they are measurable activities that are appropriate for the setting in which they are used. A recent criteria-based audit conducted in Uganda demonstrated that improvements in the quality of obstetric care can be achieved with little expense [8]. Obstetric care of high quality is a prerequisite for low maternal morbidity and mortality which require commitments of care providers and the hospital managements [8,9]. There are three types of medical audits namely case reviews/case-note presentations, national level confidential enquiries and CBA.

At Muhimbili National Hospital (MNH) in Dar es Salaam, Tanzania, up to 30% of maternal deaths are attributed to eclampsia, and eclampsia is associated with a fivefold increase in perinatal mortality [10]. Therefore, the aim of this study was to introduce of criteria-based audit to assess the quality of care among eclampsia patients admitted at a tertiary referral hospital in an African setting, and discuss possible interventions as a basis to improve the quality of care.

This paper describes the first, second and third steps of the audit cycle, i.e. identification of criteria of best practice and applying the set standards against the current practice to identify areas that are in need of improvement. The next step will be intervention to facilitate evidence-based obstetrical practice at MNH and to evaluate improvements made on the management of patients.

Ethical clearance for this work has been granted by the ethical committee of Muhimbili University of health and allied sciences, Dar es Salaam Tanzania.

## Methods

### Setting

We performed a criteria-based clinical audit of all patients diagnosed and admitted with eclampsia to the semi-intensive care unit for obstetric patients at Muhimbili National Hospital (MNH), between 14 April and 31 December 2006. This is a teaching hospital for Muhimbili University College of Health Sciences and one of four large consultant hospitals in the United Republic of Tanzania. It is situated in Dar es Salaam, the largest city with a population of about 2.5 million (45.9% females) and an annual population growth rate of 4.3% [11]. It serves as a referral hospital for the city of Dar es Salaam and the neighbouring costal region. Annual number of deliveries ranges from 10,000 to15, 000 or 25–40 deliveries per day. There was only one operating theatre for obstetrics cases due to ongoing hospital renovations during the study period and the three municipal hospitals had limited resources for 24 hours emergency operative delivery, thus most of the patients requiring surgery were referred to MNH.

The department of obstetrics and gynaecology had currently 24 specialists obstetricians and 10 postgraduate doctors working in the maternity block (10 specialists are academic staff). There are always a specialist and two postgraduate doctors on call in the maternity ward. In addition, there are two nurse officers, two nurse midwives, and two nurse assistants.

A semi-intensive eclampsia care unit was located within the labour ward with one nurse officer and two midwives on call. Patients who developed eclampsia or severe pre-eclampsia were admitted directly to the eclampsia unit. The unit manages approximately 700 (≈5% of all deliveries) patients annually.

### Guidelines for management of eclampsia at MNH

On admission, convulsions should be controlled with intravenous magnesium sulphate, airways and intravenous lines should be secured and Foley's catheter inserted into the bladder. Further convulsions after initiation of treatment should be controlled with an intravenous bolus of 2 grams of magnesium sulphate. Hypertension (systolic/diastolic blood pressure ≥160/≥110 mmHg) should be controlled with intermittent injections of hydralazine.

Baseline investigations include complete blood cell count, serum urea and creatinine, blood sugar, liver function test and, platelet count as indicated. Patient's airway should be secured. As soon as the patient is stable, labour is induced or augmented according to hospital protocols. Caesarean section under general anaesthesia should be performed when there is an obstetrical indication. After delivery, patients should be kept under close monitoring and infusion of magnesium sulphate should continue for 24 hours since last seizure or delivery depending on which occurred last. A fully recovered and ambulant patient is transferred to the obstetric wards where care continues until discharge.

### Audit procedure

The audit was performed following a criteria-based audit framework [12]. Clinical audit is the systematic and critical analysis of the quality of medical care, including the procedures used for diagnosis and treatment, the use of resources and the resulting outcome and quality of life for the patient. It follows this cycle of events:-

**Figure 1:**
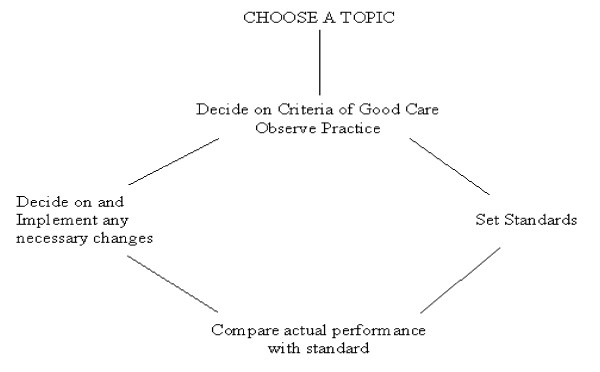
Audit Cycle

Figure 1: Audit cycle

#### Step no 1

In March 2006 a meeting was convened in the department with all doctors and nurses/midwives to agree on evidence-based criteria on management of eclampsia using the Ministry of Health (MOH) guidelines [13], local management guidelines, the WHO manual [14] supplemented by the WHO Reproductive Health Library CD-ROM no.8, standard textbooks, the Cochrane database and reviews in peer reviewed journals. A set of 14 standards (Additional file 1: Appendix 1) was developed which addressed quality issues related to the management of eclampsia. The standards were set according to the prevailing local setting and according to available resources. Two workshops were conducted to inform the department members on the standards, each workshop involved 50 doctors, nurses, and midwives.

#### Step no 2

The aim of this step was to evaluate the current practice. Data were collected by a medical doctor (senior resident) trained for the purpose. Socio-demographic data were collected by interviewing the relative accompanying the patient using a structured questionnaire. Case files were reviewed and compared against the standards, comprehensive information like antenatal care, events during antepartum and intrapartum period as well as pregnancy outcome was obtained by the principal investigator from case notes and summary. Furthermore, information was collected on gestational age at delivery, number of eclamptic seizures, blood pressure on admission, proteinuria at the antenatal clinic and on admission, gestational age at diagnosis of eclampsia, use of antihypertensive drugs, delivery complications, mode of delivery, Apgar score, birth weight, time interval between admission and delivery, and perinatal and maternal morbidity and mortality. Mothers were followed up till discharge and all babies referred to the neonatal ward were followed up for seven days to collect data on early neonatal deaths, and the causes of neonatal deaths. Cause of death was selected from the case notes as recorded by the doctor who certified the death. A post-mortem examination was not performed. The admission book was used to check if all admitted cases were included.

#### Step no 3

The results of the first audit were presented to members of the department at a specially convened unit meeting and recommendations for improvement were made.

### Statistical methods

Data were computerised using Epi Info and analysed using SPSS. Chi-square test and Fishers' exact t-test were used to compare the extent to which standard criteria were met in maternal death and survivors.

### Definitions

*Early neonatal death *= Death <7 days of life

*Birth asphyxia *= Apgar score <7 at 5 minutes after birth with cardio respiratory and neurological depression

*HELLP syndrome *= A clinical entity of preeclampsia associated with Haemolysis, Elevated Liver Enzymes and Low Platelets

*Eclampsia *=. Convulsion >24 weeks of pregnancy with hypertension and proteinuria

*Preterm delivery *= Delivery <37 completed weeks of pregnancy

*Gestational age *at delivery = Calculated according to Naegele's formula as the duration of the pregnancy in weeks, i.e. from the first day of the last normal menstrual period to the date of delivery. Ultrasound records were also used for those who had a first or early second trimester scan.

## Results

There were 389 cases of eclampsia among 7667 deliveries during the study period. The typical case of eclampsia was a young primiparous woman (Table 1). Out of 389 cases of eclampsia, 184 (47%), 159 (41%) and 46 (12%) had antepartum, intrapartum and postpartum eclampsia, respectively. Most cases of antepartum eclampsia were preterm (73%) whereas intrapartum (61%) and postpartum (71%) eclampsia more often developed at term. A median of two seizures had occurred before admission (Range: 1–12). The hospital-based incidence of eclampsia was 504 per 10,000 women or 5.1% of all mothers admitted.

**Table 1:** Basic characteristics of 389 women diagnosed with eclampsia

Basic characteristic	Eclampsia							
			
	Antepartum (n = 184)		Intrapartum (n = 159)		Postpartum (n = 46)		Total (n = 389)	
				
	n	%	n	%	n	%	n	%
**Age (years)**:								
15–24	125	68	104	65	33	72	262	67
25–34	43	23	44	28	7	15	94	24
35–45	8	4.3	4	2.5	5	11	17	4.4
Unknown	8	4.3	7	4.4	1	2.2	16	4.1
**Parity:**								
0	122	66	106	67	32	70	260	67
1–2	52	28	41	26	13	28	106	27
3–7	10	5.4	12	7.5	1	2.2	23	5.9
**Number of antenatal visits**:								
0	14	7.6	11	6.9	6	13	31	7.9
1–2	79	43	54	34	19	41	152	39
≥3	91	50	94	59	21	46	206	53
**Induction**	184	100	8	5.0	-	-	192	49
**Mode of delivery**:								
SVD*	145	79	104	65	29	63	278	72
CS*	32	17	39	25	5	11	76	20
LCVE*	1	0.5	1	0.6	-	-	2	0.5
ABD*	4	2.2	3	1.9	1	2.2	8	2.1
Others	1	0.5	10	6.3	1	2.2	12	3.1
Unknown	1	0.5	2	1.3	10	22	13	3.3

*SVD = Spontaneous vertex delivery, CS = Caesarean section, LCVE = Low cavity vacuum extraction, ABD = Assisted breech delivery; 23% of patient delivered by CS were initially had induction of labour

### Antenatal care

The median number of antenatal visits was 3 (Range: 0–12) but only 24% had a diagnosis of pre-eclampsia during antenatal care prior to convulsions. A third of all maternal deaths occurred among the 7.9% of the mothers who did not attend antenatal care. Proteinuria was not assessed at the antenatal clinic in 55% of cases.

### Mode of delivery

The majority of the mothers had spontaneous vertex delivery (72%), and the caesarean section rate was 20% (Table 1). The proportion of preterm deliveries by caesarean section and spontaneous vertex was 29% and 58%, respectively. For those who were not delivered before arrival the median time interval between admission and delivery was 10 hours (Range: 10 minutes-90 hours) and one third were not delivered within 24 hours.

### Gestational age and birth weight

The median gestational age at delivery was 36 weeks (Range: 24–42); 14% of were delivered before 32 weeks and 54% (n = 209) 37 weeks. The mean birth weight for a singleton baby was 2247 grams. The prevalence of low birth weight and very low birth weight was 32% and 18%, respectively (Table 2).

**Table 2:** Foetal and maternal outcomes among women diagnosed with eclampsia

Foetal and maternal outcome	Eclampsia							
			
	Antepartum (n = 184)		Intrapartum (n = 159)		Postpartum (n = 46)		Total (n = 389)	
				
	n	%	n	%	n	%	n	%
**Gestational age at delivery (weeks**)								
24–32	28	15	25	16	2	4.3	55	14
32–36	79	43	59	37	16	35	154	40
37–42	76	41	75	47	27	59	178	46
Unknown	1	0.5	-	-	1	2.2	2	0.5
**Apgar score at 5 min:**								
0	50	27	40	25	11	24	101	26
1–2	7	3.8	9	5.7	1	2.2	17	4.4
3–6	45	25	38	24	4	8.7	87	22
≥7	81	44	67	42	14	30	162	42
Unknown	1	0.6	5	3.1	16	35	22	5.6
**Foetal outcome**:								
MSB*	16	23	9	14	-	-	25	6.4
FSB before admission*	14	20	15	24	5	18	34	8.7
FSB after admission	19	27	7	11	-	-	26	6.7
Early neonatal death	19	27	23	37	3	11	45	12
Unknown	2	2.8	9	14	20	71	31	7.9
**Number of newborn**:								
Singleton	176	96	131	82	39	85	346	89
Twins	8	4.4	24	15	2	4.3	34	8.7
Triplets	-	-	-	-	3	6.5	3	0.8
Unknown	-	-	4	2.5	2	4.3	6	1.5
**Birth weight (grams)**:								
500–1499	39	21	27	17	6	13	71	18
1500–2499	69	38	47	30	8	17	124	32
2500+	75	41	80	50	28	61	183	47
Unknown	1	0.5	5	3.1	4	8.7	11	2.8
**Maternal outcome**:								
Alive	177	96	145	91	38	80	359	92
Dead	7	3.8	14	8.8	9	20	30	7.7
Unknown	-	-	-	-	-	-	1	0.2

*FSB = *Fresh still birth*, MSB = *Macerated stillbirth*

### Perinatal and maternal morbidity and mortality

There were 30 maternal deaths and in 40% their babies also died (Table 2). Out of 161 perinatal deaths, 37% were foetal deaths before admission, 16.1% of the foetal death occurred after admission or during labour, and 28.9% were early neonatal deaths, 19 were unclassified due to poor documentation. In, 63 (16%) mothers there was an additional major complication (Table 3). Cerebral vascular haemorrhage had the highest case fatality rate (80%) followed by pulmonary oedema and respiratory failure (53%). The case fatality rate was 7.7% and the perinatal mortality rate was 214 per 1000 live births (table 3). At the closure of data collection two patients were still in the ICU due to irreversible complication of massive brain haemorrhage.

**Table 3:** Case fatality rate (CFR) in women diagnosed with eclampsia by major complication

Complication	All cases	Fatalities	CFR
Cerebrovascular haemorrhage	15	12	80
Pulmonary oedema/respiratory failure	17	9	53
HELLP* syndrome	4	1	25
Post partum haemorrhage	17	4	24
Aspiration pneumonia	5	1	20
Abruption of the placenta	7	1	14
Uterine rupture	7	1	14
Acute renal failure/uraemia	7	1	14
Sepsis	4	0	0
No major complication	306	-	-

Total	389	30	7.7

*HELLP = Haemolysis, Elevated Liver Enzymes and Low Platelets

### Substandard care

Table 4 summarizes care given to eclampsia patients as compared to standard. Suboptimal care was mainly on management plan by senior staff, review of the plans by specialist obstetrician. Prompt surgical delivery, assessment of deep tendon reflexes and laboratory tests.

**Table 4:** Adherence to audit standards on the management of women diagnosed with eclampsia at MNH.

Audit criteria	Eclampsia					
	Antepartum (n = 184)		Intrapartum (n = 159)		Postpartum (n = 46)	
	n	%	n	%	n	%
Detailed history and documentation	174	95	151	95	46	100
Management plan by senior personnel.	132	72	102	64	7	72
Use of MgSO_4_.	184	100	159	100	46	100
Treatment of severe hypertension	105	57	110	69	30	65
Management plans review within 2 hours of admission by a specialist obstetrician	47	25	41	26	11	24
Blood pressure measurement at least every half an hour	176	96	142	90	38	83
Urine analysis for proteinuria within 2 hours of arrival	120	65	90	57	26	56
Fluid balance chart should be maintained for 48 hours,	182	99	157	99	46	100
Deep tendon reflexes test to all patients treated with MgSO_4_	0	0	2	1.3	0	0
Respiration rate monitored for 24 hours in all patients treated with magnesium sulphate	184	100	159	100	46	100
Use of corticosteroids for lung maturation	*2/77	2.6	*1/51	2	-	-
Caesarean section) within 2 hours of decision	*10/37	27	*14/39	36	-	-
Delivery should be within 24 hours	127	77	117	91	-	-
Full blood count to all admitted patient	52	29	43	27	13	28
Serum Urea and creatinine to all admitted patient	70	38	60	38	17	37
Liver function test (liver enzymes) to all admitted patient	7	3.8	3	1.9	4	9
Proper use of the parthogram and monitoring	*166/181	92	*93/136	68	-	-
Prompt and early referral	177	96	137	86	38	83

(*cases less than total n, for example women with premature labour, cases admitted before delivery, cases delivered by CS and SVD).

All patients with seizures were treated with magnesium sulphate, but only two patients had deep tendon reflexes assessed. Delay in operative delivery was common. Two out of three patients requiring operation were not operated within set standards. Out of 132 patients with preterm labour only 3 were given corticosteroids for foetal pulmonary maturation. Patients admitted during day time were more likely to be reviewed by specialists than those admitted at night (Table 5).

**Table 5:** Time of admission against time the patient was reviewed by a specialist obstetrician

Characteristic	Time seen by a specialist				Number of women
		
	Within 2 hours	Within 3–6 hours	After 6 hours	Not seen	
*Time of admission to the eclampsia room (hours*)					
00.00–07.59	19	19	29	23	90
08.00–13.59	28	30	24	17	99
14.00–19.59	31	20	25	24	100
20.00–23.59	17	36	19	28	100
*Survival status*					
Maternal death	15	10	3	2	30
Survivor	80	95	94	90	359

Number of women	95 (24%)	105(27%)	97 (25%)	92 (24%)	389 (100%)

### Source of admission

Out of 389 eclampsia patients admitted to MNH 291 (65%) were referred from neighbouring municipal hospitals and out of these 26 (8.7%) had already delivered.

## Discussion

This study evaluated the hospital care of patients with eclampsia in a low-income country and revealed several suboptimal factors that must be corrected in order to improve maternal and foetal outcomes. Most of these improvements can probably be performed without extra monetary input. This was the first attempt to identify areas of weakness in the management of eclampsia patients in this urban tertiary centre. In this stage of the audit cycle our main task was to evaluate the current practice against set standard, the next level of the audit will assess the practice after intervention.

The importance of audit and the collection of process indicators were highlighted at the Safe Motherhood Technical Consultation in Colombo in 1997 and criteria-based audit is a proven method to improve the quality of maternity care [15,16]. An audit may play a dual role, not only to monitor change in support of clinically effective practice but also as an educational tool. It is part of a process for improvement and can empower health workers to conduct their own quality assessments and seek their own solutions that are locally appropriate and internally driven [16].

The main areas of care which were suboptimal in terms of the set standards were mainly inadequate medical history, lack of review of management plans by specialists, not delivered within 24 hours, and poor monitoring during labour. In this centre the medical history is recorded by junior doctors (interns, junior residents) but the management plan is the responsibility of a more senior doctor (senior residents and specialists), Although a quarter of the patients were reviewed by specialist it did not make any difference since the specialist were most often called for very sick patients, however, this study did not evaluate the condition of the patient at the referring hospital prior to referral. Eclampsia is such a potentially dangerous condition that need comprehensive care in order to prevent both maternal and foetal morbidity and mortality [17], hence, a specialist assessment of all patients is mandatory irrespective of their condition on admission. The majority of the specialist doctors live far from the hospital and do not spend nights in hospital even when they are on duty. Therefore, to improve the standards of care in this urban tertiary centre as a long term solution the hospital management should create an environment to enable the specialist to spend nights at the hospital when on call. However, for an interim solution junior medical personnel (doctors/nurses) should be trained (continued medical education) to be able to effectively manage these patients under close supervision by senior obstetrician.

Despite access to a well-equipped laboratory the majority of the patients were not properly investigated, hence, early diagnosis of serious morbidity and mortality such as the HELLP syndrome [17,18] was impossible. In contrast to studies from developed countries renal failure and coagulation problems were relatively rare in the current study, only severe cases were detected. This might be due to inadequate use of laboratory to detect impaired renal function as indicated by fewer patients having test for serum urea and creatinine.

Pregnant women in Tanzania have free medical care, therefore, there was no cost for laboratory test. Lack of conducting these tests may reflect the inadequacy of current protocols or lack of knowledge among health care providers on the pathophysiology of eclampsia.

This audit shows the positive effect of magnesium sulphate as indicated by few recurrent seizures after initiation of the treatment, however, lack of deep tendon reflex test was a serious omission. This is an important test, since the evidence shows that magnesium toxicity is monitored by appropriate clinical monitoring [19] not necessarily magnesium serum levels. Some of the women with recurrent seizures might have needed an increased dose while others might have needed a reduced dose in order to avoid toxicity. Magnesium toxicity may result in coma, cardiac or respiratory arrest [19,20]. This finding might have been due to lack of coordination between doctors and nurses or lack of documentation. This assessment should normally be done by either a nurse or a doctor.

The most common reasons for maternal deaths were cerebral haemorrhage and pulmonary oedema. Both these complications should be possible to prevent through more adequate control of the blood pressure and fluid balance. Intermittent positive pressure can also be used to treat pulmonary oedema until the pathology resolves, however, this service was not available in the study area. There is a need to have adequately equipped intensive care units in tertiary centres for treatment of severe preeclampsia and eclampsia in order to reduce maternal and foetal morbidity and mortality.

Preterm delivery and low birth weight are well known major causes of perinatal mortality [21]. Few preterm mothers received corticosteroids in this study, maybe because most of them were referred and had spent considerable time at the referring hospital, and therefore were delivered as soon as they were admitted to MNH. The high prevalence of low birth weight (51%) found in this study is coherent with other reports [22,23].

A major limitation of this study is that it was performed at a tertiary hospital that receives patients from other hospitals where standards of care are not known. Furthermore, the standard of care during pregnancy as well as the role of antenatal care has not been assessed. However, a previous study by Urassa et al in the same hospital indicated poor ability of antenatal care to perform even basic investigations such as proteinuria and to initiate appropriate monitoring of risk cases. As primary prevention of eclampsia cannot be expected there is a great need of effective life saving obstetric emergency care in all levels. The three municipal hospitals in Dar es Salaam accounted for 75% of all admissions and 70% of the maternal deaths in the unit. Therefore, it is not possible to achieve a major change in outcome of eclampsia unless the primary care providers play their role of early detection of the problem, provide first aid, and prompt referral to higher centres for definite management. It is necessary to streamline the referral system and have common management protocol in all referring hospitals.

## Conclusion

Potential areas for further improvement in quality of emergency care for eclampsia relate to standardizing management guidelines, greater involvement of specialists in the management of eclampsia and continued medical education on current management of eclampsia for junior staff.

## Competing interests

The authors declare that they have no competing interests.

## Authors' contributions

HLK participated in the design of the study, data collection, statistical analysis, interpretation of the data, and wrote the first draft of the paper. GL participated in the design of the study, supervised interpretation of the data, and contributed to the development of the manuscript. IM contributed to the interpretation of the data, and the development of the manuscript. SNM participated in the design of the study and supervised the data collection as well as development of the manuscript. LN participated in the design of the study, supervised the data analysis, contributed to the interpretation of the data, and the development of the manuscript.

## Pre-publication history

The pre-publication history for this paper can be accessed here:



## Supplementary Material

Additional file 1 **Selected audit criteria (standards) for the study.** Click here for file
