# Supplementary material for: Criteria-based audit on management of eclampsia patients at a tertiary hospital in Dar es Salaam, Tanzania
Source: BMC Pregnancy Childbirth. 2009 Mar 27;9:13. doi: 10.1186/1471-2393-9-13 (PMC2670267; doi:10.1186/1471-2393-9-13)
Supplement: Additional file 1 — Selected audit criteria (standards) for the study. [file 1471-2393-9-13-S1.doc]

| **Appendix 1. Audit criteria(standard)** | |
| --- | --- |
| No. | Audit standard (criteria) |
| 1 | Detailed history and documentation should be made as soon as the patient is admitted |
| 2 | Management plan should be made by senior personnel (senior residents, midwives or registrars). |
| 3 | All eclamptic patients should receive MgSO4 as treatment and prophylaxis for further seizures. |
| 4 | Treatment of severe hypertension (DBP>110mmHg)with IV medication to all patients with hypertension |
| 5 | All patients’ management plans should be reviewed within 2 hours of admission by a specialist obstetrician. |
| 6 | All patients should have blood pressure measurement at least every half an hour |
| 7 | Urinalysis for proteinuria should be done within 2 hours of admission |
| 8 | Fluid balance chart should be maintained for 48 hours, in order to monitor urine output and that no patient should be put at risk of fluid imbalance and pulmonary oedema |
| 9 | Deep tendon reflexes should be monitored in all patients treated with magnesium sulphate |
| 10 | Respiration rate should be monitored for 24 hours in all patients treated with magnesium sulphate |
| 11 | Corticosteroids for lung maturation should be given to all preterm cases |
| 12 | Operative delivery (Caesarean section) should be performed within 2 hours of decision |
| 13 | Delivery should be within 24 hours |
| 14a | Full blood count should be done at least once to all admitted patient |
| 14b | Renal function test (urea and serum creatinine) should be done at least once to all admitted patient |
| 14c | Liver function test (liver enzymes) should be done at least once to all admitted patient |
